# Enhanced antitumor effect on intrapulmonary tumors of docetaxel lung-targeted liposomes in a rabbit model of VX2 orthotopic lung cancer

**DOI:** 10.1038/s41598-017-10530-8

**Published:** 2017-08-30

**Authors:** LiJuan Wang, Rui Li, KeKe Che, ZhongHong Liu, ShiFeng Xiang, MengYa Li, Yu Yu

**Affiliations:** 1 Pharmacy College, Chongqing Medical University, Chongqing, 400016 China; 2 Department of Pharmacy, Chongqing Medical and Pharmaceutical college, Chongqing, 401331 China; 3 Department of Pharmacy, Chongqing General Hospital, Chongqing, 400014 China; 4 Radiology department, Chongqing General Hospital, Chongqing, 400014 China

## Abstract

Allergic reactions and severe systemic toxicity are two major challenges for the clinical application of docetaxel (DTX) for treatment of non-small-cell lung cancer (NSCLC). We developed a novel lung-targeted DTX-loaded liposome (DTX-LP), an efficient drug delivery system, with a patented DBaumNC technology to overcome these deficiencies. In the present study, we describe the targeting activity, tumor inhibition rate (TIR), survival, pathology, tumor apoptosis and metabolism of DTX after intravenous injection of DTX-LP compared to the DTX injection (DTX-IN) formulation based on the VX2 orthotopic lung cancer rabbit model. Biodistribution studies revealed the highest accumulation in lung and tumor within 12 h after the injection of DTX-LP. The increased TIR indicates that the growth of tumor was slowed. Pathology tests demonstrated that DTX-LP can reduce metastasis and toxicity to non-targeted organs, leading to greatly extended survival time and improved survival of tumor-bearing rabbits. Flow cytometry and immunohistochemistry confirmed that DTX-LP is highly efficacious in tumor tissue, leading to a significant increase of tumor apoptosis and decrease of proliferation and angiogenesis. The results from this study demonstrate the increased intrapulmonary tumor targeting activity, enhanced antitumor effect and reduced toxicity of DTX-LP compared to DTX-IN and highlight its clinical prospects for NSCLC therapy.

## Introduction

Lung cancer is a primary malignancy of the lungs and the leading cause of cancer-related deaths worldwide^1–3^. In 2012, approximately 1.8 million new cases of lung cancer were diagnosed and 1.6 million patients died of lung cancer. The lung cancer diagnosis (13.0%) and mortality (19.4%) rates rank first among all types of cancers. In China, specifically, the incidence and mortality of lung cancer are greater than those of other types of cancer. Approximately 80–85% of lung cancer patients have NSCLC^4^. Although the current recommendation for managing early stage NSCLC is surgery, most primary and secondary lung cancer patients receive chemotherapy and radiation treatments. Unfortunately, long-term survival outcomes for these patients remain poor^5, 6^.

DTX, a semisynthetic taxane, acts by binding to the β-subunit of tubulin, and promotes stabilization of microtubules, causing G2/M cell cycle arrest^7^. It is the only drug with demonstrated activity approved by the FDA for both first- and second-line therapy for treatment of advanced NSCLC^8, 9^. Although DTX has significant antitumor activity, its short circulation half-life, poor aqueous solubility and severe side effects often compromise its clinical efficacy^10–13^. At present, there is only one formulation for intravenous injection on the market (DTX-IN) which contains polysorbate 80 as solubilizer. Allergic reactions and signs of severe systemic cytotoxicity, such as myelosuppression, neuropathy, diarrhea, stomatitis, nausea, and vomiting are major challenges for the clinical application of DTX injection in therapy of NSCLC. Studies suggest that many of these adverse effects are largely attributed to non-selective distribution and poor organ selectivity after intravenous administration *in vivo* ^14^. Therefore, the focus for improving the treatment of NSCLC with DTX is to increase its concentration in intrapulmonary tumors. Selective distribution in lung tumors should enhance the antitumor effect of DTX and decrease its toxicity^15^.

It has been reported that new formulations of DTX, such as nanoparticles^16^, intravenous lipid emulsions^17^, nanostructured liposomes^18^, microemulsions^19^, chitosan-anchored liposomes^20^ and PEGylated immunoliposomes^21^, may increase the efficacy of targeted delivery to specific tumor cells without obvious toxic side effects. However, few studies have shown whether the accumulation of DTX was increased in intrapulmonary tumors, specifically. Meanwhile, there are still barriers for those formulations to meet the need for clinical use and industrial production.

To overcome these deficiencies, a novel formulation of DTX, DTX-LP, was designed with patented DBaumNC technology by combining the solid dispersion and effervescent techniques from our own laboratory^22, 23^. The produced liposomes are at approximately 1 μm in diameter and stable at various conditions. It was previously confirmed that DTX-LP significantly accumulated in lungs^24^. The purpose of this study was to evaluate targeting activity, TIR, survival, pathology, and tumor apoptosis after intravenous injection of DTX-LP compared to the DTX-IN (Texotere®) in the VX2 orthotopic lung cancer rabbit model. Additionally, the distribution of DTX in lung tumors after intravenous administration of DTX-LP and DTX-IN was measured to evaluate the lung-tumor-targeting profile. Change in tumor size and TIR was calculated by CT imaging. The tumor apoptosis, proliferation, and angiogenesis were measured by flow cytometry and immunohistochemistry. Pathology of all tissues, including tumors, was used to evaluate organ toxicity and tumor necrosis. DTX and its metabolites in feces samples were analyzed for investigation of metabolic changes by UPLC-MS/MS.

## Results

### Zeta potential, size distribution, encapsulation efficacy and the drug loading of DTX-LP

**Figure 1:**
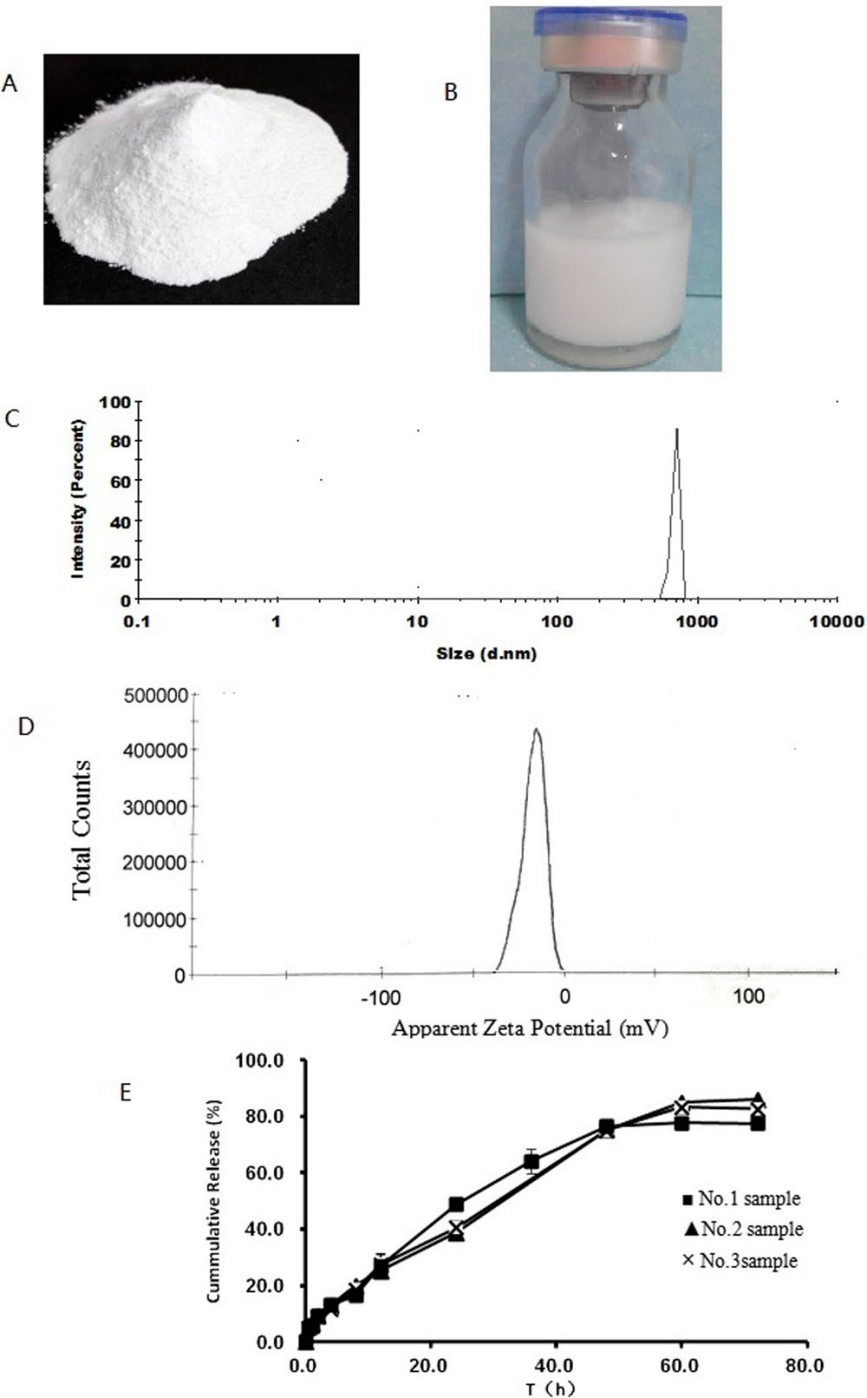
The characterization of DTX-LP. (**A)** The DTX-LP solid dispersion (**B)**. The DTX-LP suspension (**C**). The diagram of size distribution of DTX-LP (**D**). The diagram of zeta potential distribution of DTX-LP E. The cumulative release curve of DTX-LP *in vitro*.

The DTX-LP solid dispersion was first prepared with patented DBaumNC technology (Fig. 1A). Before administration, the DTX-LP solid dispersion was made into suspension which was a uniform emulsion from the appearance by effervescent techniques (Fig. 1B). We characterized the prepared DTX-LP suspension formulation. The size distribution of DTX-LP was between 500 nm and 800 nm. The average size of three samples was 682.6 ± 148.5 nm (Fig. 1D). The zeta potential of DTX-LP was −16.4 ± 1.5 mV (Fig. 1C). The encapsulation efficacy and the drug loading were 88.87% ± 1.24% and 6.55 ± 0.08 mg/g by HPLC assay. The cumulative release curve of DTX-LP *in vitro* was shown in Fig. 1E. The cumulative release rate after 60 h was almost 80%.

### Minimally invasive percutaneous puncture inoculation to establish VX2 orthotopic lung cancer rabbits model

**Figure 2:**
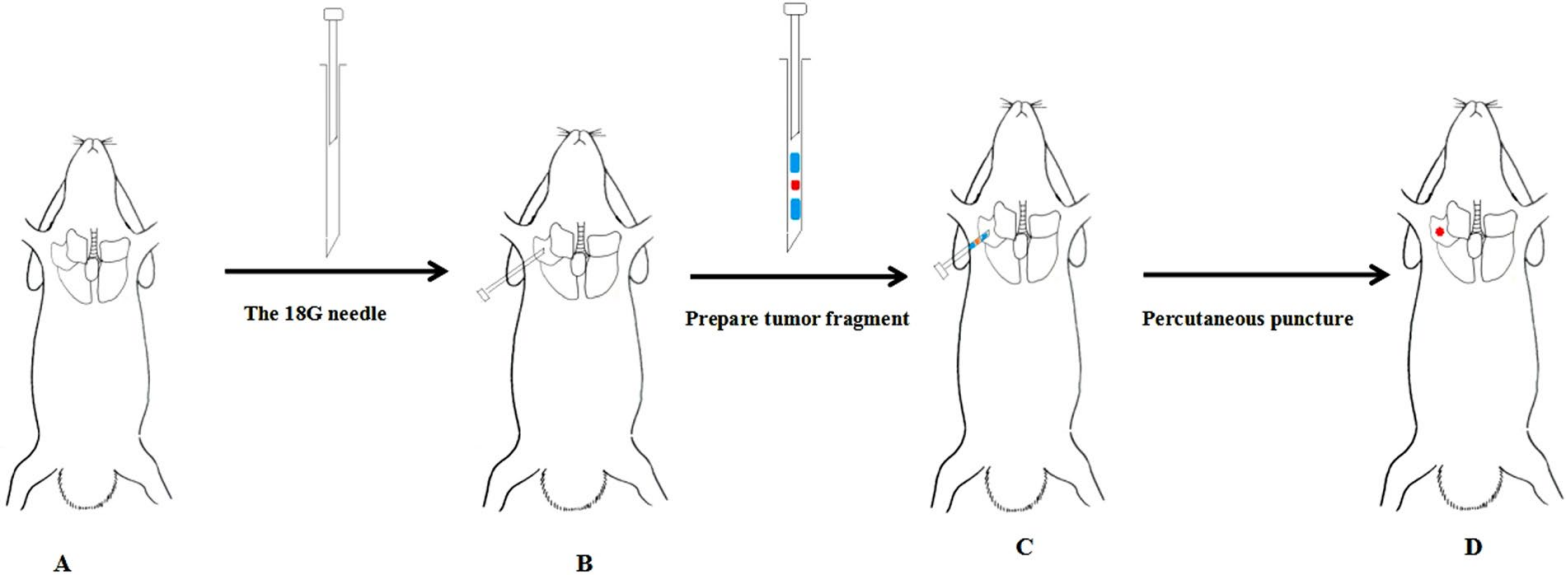
The procedure of minimal invasive percutaneous puncture inoculation to establish a VX2 orthotopic lung cancer rabbits model. (**A**) Fixed on the digital gastrointestinal machine; (**B**) position *in vitro* (18-gauge needle was prepared before inoculation, blue: 0.5 cm gelatin sponge, red: 1 mm^3^ tumor fragment); (**C**) position *in vivo*; (**D**) the probable position of tumors (confirmed by Fig. 2).

**Figure 3:**
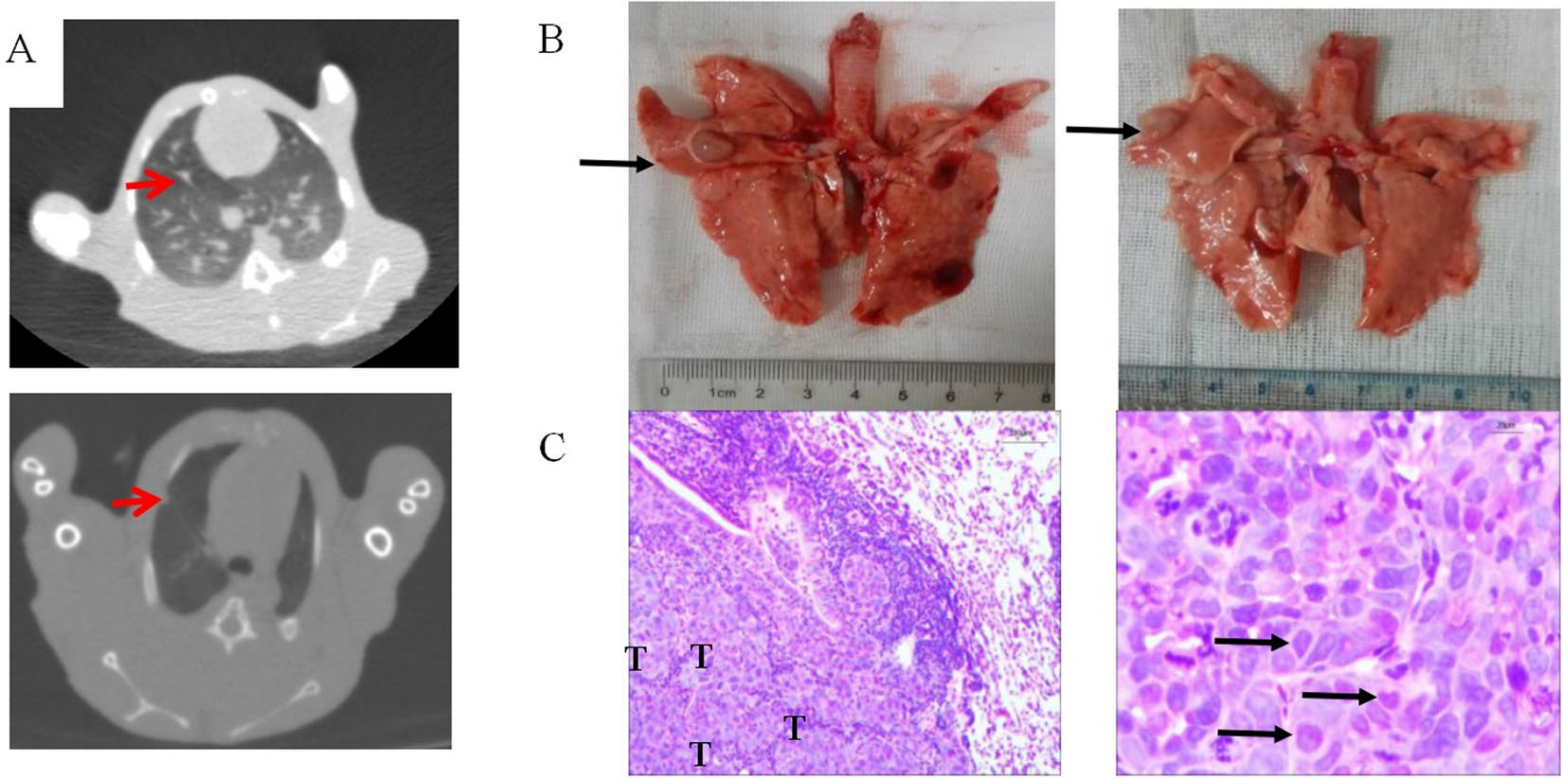
The confirmation of VX2 orthotopic lung cancer rabbits model. (**A**) CT scanning (red arrow means the single and localized intrapulmonary tumor) (**B**) Necropsy (black arrow means the single and localized intrapulmonary tumor) (**C**) HE pathological examination (left: 100×, right: 400×, “T” means tumor cell, black arrows mean nuclei with hyperchromatism and pleomorphism).

The procedure is shown in Fig. 2. Two weeks post-inoculation of VX2 tumor fragment, single and localized intrapulmonary tumors that were 2–6 mm in diameter were observed on CT images, which grew as ellipsoidal or irregular circular nodes (Fig. 3A). Nodes in lungs were ovoid or irregularly shaped, soft tissue with a clear boundary from the pulmonary window and showed a spot from the mediastinal window with a CT value of 28 HU. Analysis of the anatomy from CT images showed that the VX2 transplanted tumor nodules in rabbits were hoary and localized in the right lung (Fig. 3B). HE staining revealed that the tumor was poorly differentiated squamous cell carcinoma (Fig. 3C). Under low power, disorderly growth of the squamous epithelial cells in large nests was visible. At high power, the tumor cells revealed crowded nuclei with hyperchromatism and pleomorphism, such as circular, ovoid, and cubic shapes. Pathological nuclear division was also observed. Tumor seeding in the chest wall was scarce using the minimally invasive percutaneous puncture inoculation.

### CT imaging and TIR

**Figure 4:**
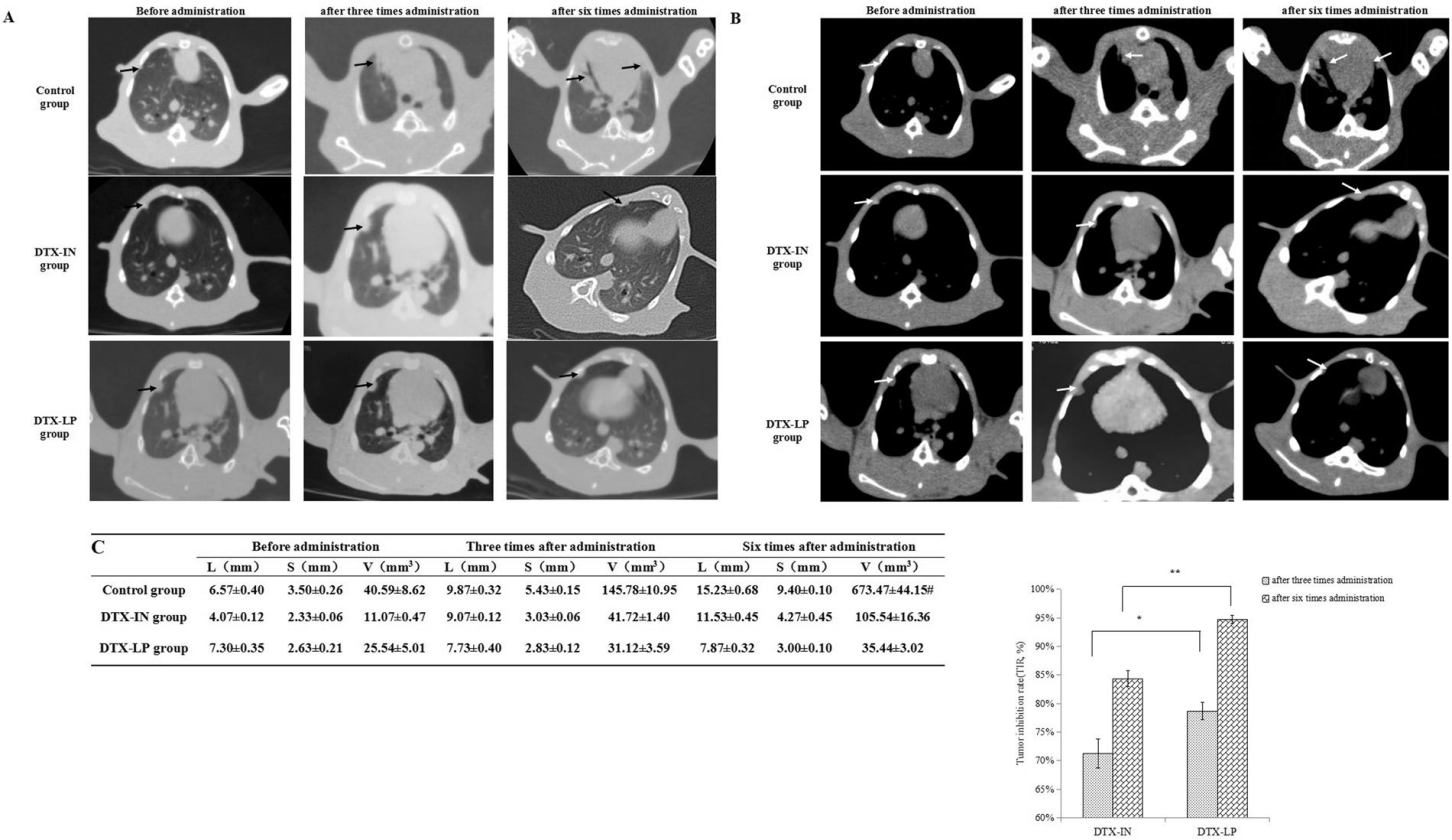
CT imaging. (**A**) Pulmonary window (black arrow means the single and localized intrapulmonary tumor), (**B**) Mediastinal window (white arrow means the single and localized intrapulmonary tumor); (**C**) the volumes of tumors (mm^3^) calculated from CT imaging measured by experienced technician (n = 3, ^#^the tumor had been spread to the chest), (**D**) TIR (%) calculated by formula previously described (t-test, *p* ≤ 0.05) *p < 0.05, **p < 0.01.

Figure 4A and B show CT images of control, DTX-IN, and DTX-LP groups before administration and after three and six weekly administrations. The tumor volumes in DTX-IN and DTX-LP groups were significantly smaller than those in control group, and tumor volumes in DTX-LP group were smaller than those in the DTX-IN group (Fig. 4C). These observations indicated that tumor growth was significantly suppressed by DTX-LP. Furthermore, the TIRs were calculated (Fig. 4D). After three weekly treatment administrations, the TIRs (%) of DTX-IN and DTX-LP groups were 71.27% ± 2.54% and 78.68% ± 1.41%, respectively (*p* < 0.05). The TIRs (%) of DTX-IN and DTX-LP groups after six weekly administrations were 84.39% ± 1.48% and 94.71% ± 0.75%, respectively (*p* < 0.01).

### HPLC assay to detect distribution of DTX in tumors

**Figure 5:**
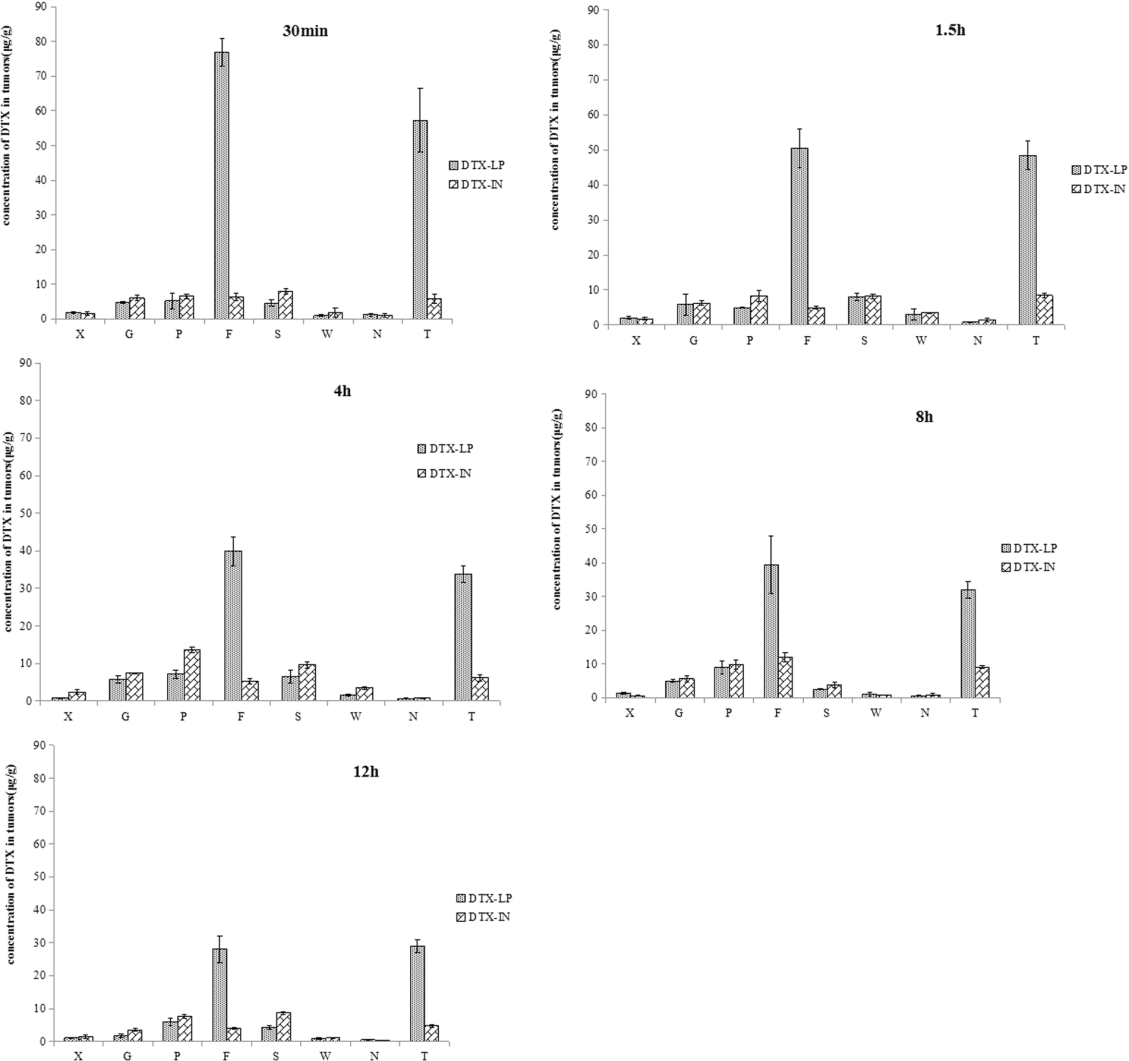
Distribution of DTX in tissues by HPLC assays at different time points after administration of DTX-IN and DTX-LP (µg/g, Mean ± SD, n = 3). (**A**–**E**) Means 30 min, 1.5 h, 4 h, 8 h and 12 h; “X, G, P, F, S, W, N, T” means heart, liver, spleen, lungs, kidneys, stomach, brain and tumor.

The distribution of DTX in tumors at different time points after the same dose of DTX-LP and DTX-IN was significantly different (Fig. 5). The concentrations (in μg DTX/g tissue) at 30 min, 1.5 h, 4 h, 8 h, and 12 h were, respectively, 76.97 ± 3.99, 50.45 ± 5.46, 39.87 ± 3.89, 39.42 ± 8.52, 28.03 ± 4.10 μg/g in lung tissue and 57.31 ± 9.30, 48.48 ± 4.21, 33.80 ± 2.23, 32.05 ± 2.37, 28.93 ± 2.01 μg/g in tumor tissue for the DTX-LP group. For the DTX-IN group, concentrations of DTX at 30 min, 1.5 h, 4 h, 8 h, and 12 h were, respectively, 6.36 ± 1.06, 4.96 ± 0.29, 5.30 ± 0.84, 12.11 ± 1.40, 3.89 ± 0.28 μg/g in lung tissue and 5.83 ± 1.39, 8.43 ± 0.66, 6.20 ± 0.85, 9.16 ± 0.30, 4.72 ± 0.50 μg/g in tumor tissue. The concentrations of DTX in lung, and tumor tissues were significantly increased when the DTX was prepared as the lung-targeted liposome formulation. Therefore, it follows that antitumor efficacy is improved with the DTX-LP formulation.

### Tumor proliferation and apoptosis by TUNEL assay, PCNA assay and flow cytometry

**Figure 6:**
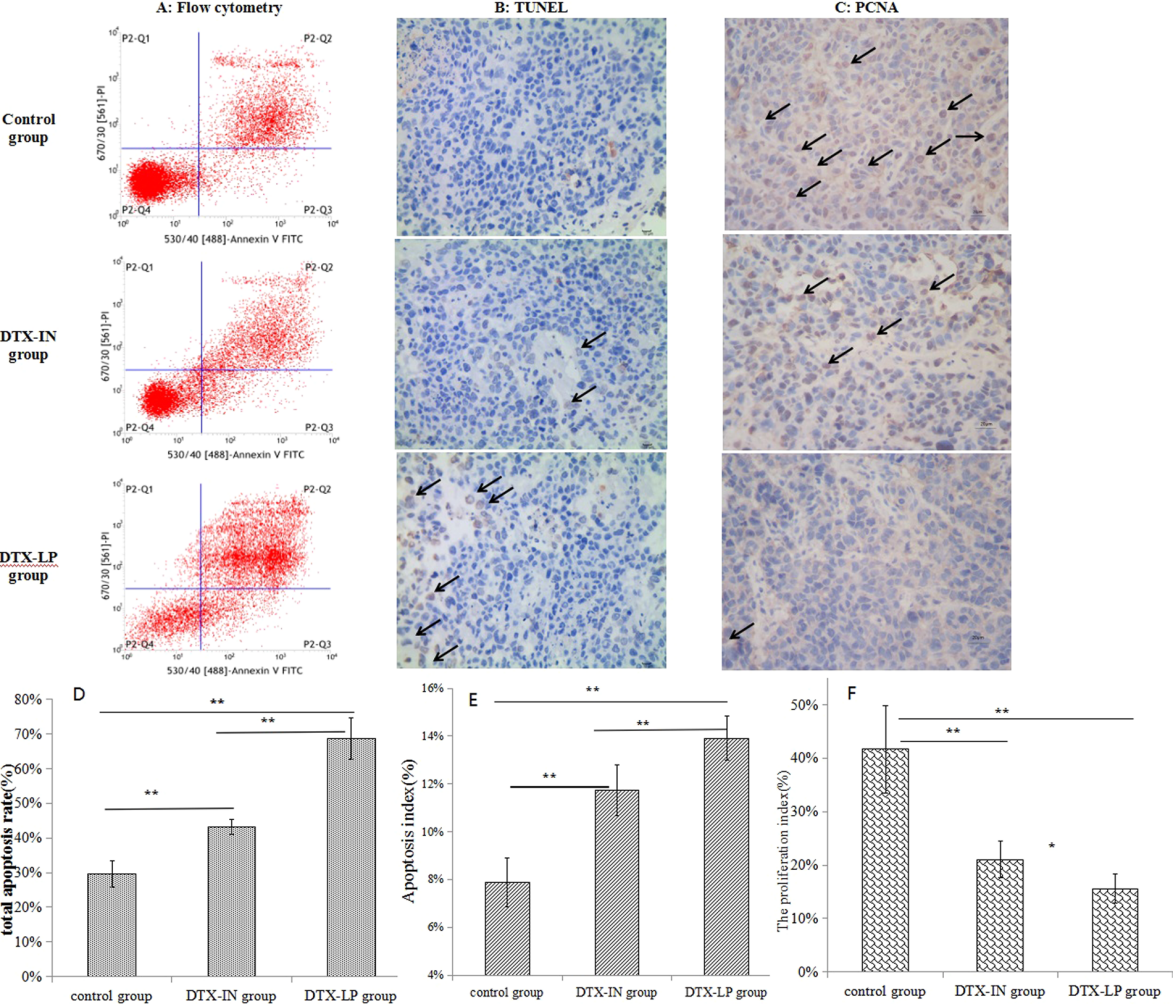
The proliferation and apoptosis in tumors. (**A**,**D**) Flow cytometry assay of tumor apoptosis within tumor tissues after treatment with DTX-IN, DTX-LP or saline. The number of apoptosis cells included early and late apoptosis cells. (**B**,**E**) Apoptosis measurement after treatment with DTX-IN, DTX-LP or saline by TUNEL assay. Number of TUNEL-positive cells counted from randomly selected field of each tumor section (Black arrow means TUNEL-positive cells). (**C**, **F**) PCNA analyses of cancer cell proliferation within tumor tissues after treatment with DTX-IN, DTX-LP or saline. Number of PCNA-positive cells counted from randomly selected field of each tumor section (Black arrow means PCNA-positive cells). Each bar represents the mean percentage proliferation or apoptosis ± SD (n = 6). *p < 0.05, **p < 0.01, (t-test, *p* ≥ 0.05).

Tumor proliferation and apoptosis in VX2 orthotopic lung cancer rabbits were confirmed by TUNEL, PCNA assay and flow cytometry after treatment with DTX-IN, DTX-LP, or saline (Fig. 6). Tumors treated with DTX-LP had higher total apoptotic rate than tumors treated with the alternative DPX formulation (Fig. 6A and D). The apoptotic tumor index from VX2 orthotopic lung cancer rabbits treated with DTX-LP was significantly greater than that of the rabbits that received DTX-IN, and this index was two times greater than that of the control group (Fig. 6B and E). Compared to the DTX-IN group, animals that received DTX-LP had significantly less tumor cell proliferation (Fig. 6C and F). The proliferation index of tumors treated with DTX-LP was more than three times less than the proliferation index of the control group.

### Tumor angiogenesis by CD31 assay

**Figure 7:**
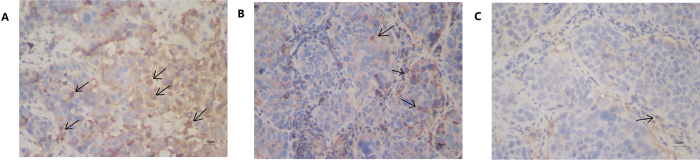
The CD31 immunohistochemistry of tumor sections of VX2 lung cancer rabbits after injection with saline, DTX-IN and DTX-LP after six times administrations. (**A**) Control group; (**B**) DTX-IN group; (**C**) DTX-LP group. Black arrow means the CD31-positive blood vessels which have been stained brown.

The CD31 assay was used to evaluate the degree of tumor angiogenesis, which indicates a rapidly growing tumor (Fig. 7). In the DTX-LP group, the CD31-positive blood vessels were smaller than those in the DTX-IN and control groups. The weaker staining signal in the DTX-LP group suggests that the DTX-LP treatment had greater antitumor activity, presumably due to increased accumulation of DTX in tumor tissues.

### Necropsy and pathology test in tumors

**Figure 8:**
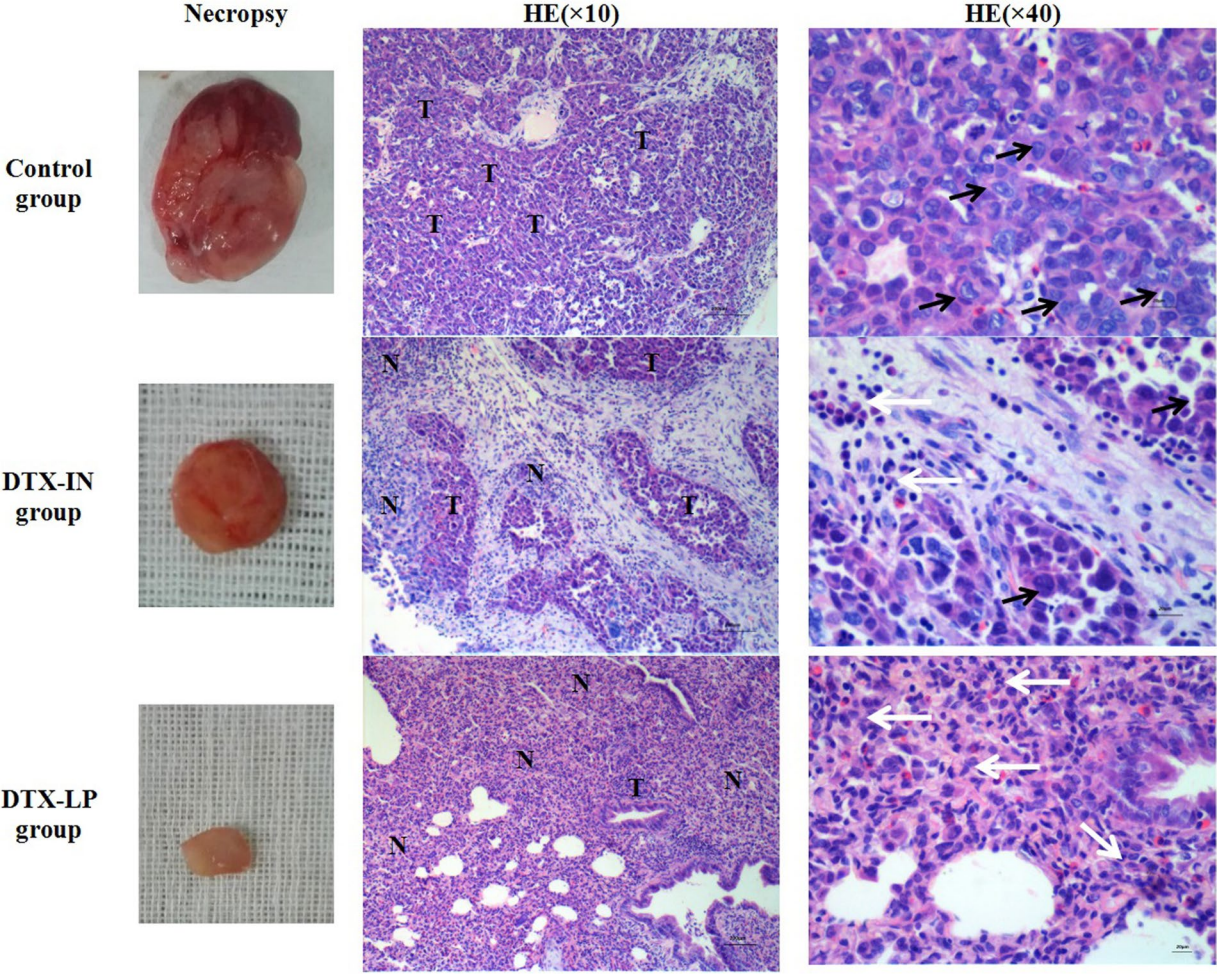
Result of pathological test (HE staining) of tumors after six times administrations in each group (“T” shows tumor, “N” shows necrosis, black arrows show tumor cells, white arrows show inflammatory cells).

HE staining of tumors revealed the degree of necrosis and tumor cell morphology (Fig. 8). Observation of the entire set of tumor sections for the three treatment groups revealed differences in cellular features with varying degrees of tumor cell necrosis. The control group showed less necrosis and more pleomorphic nuclei than the other two groups. Tumor sections from the DTX-IN group showed moderate necrotic regions with inflammatory cells present. The DTX-LP group showed extensive necrosis, and a few tumor cells were observed.

### Survival analysis

**Figure 9:**
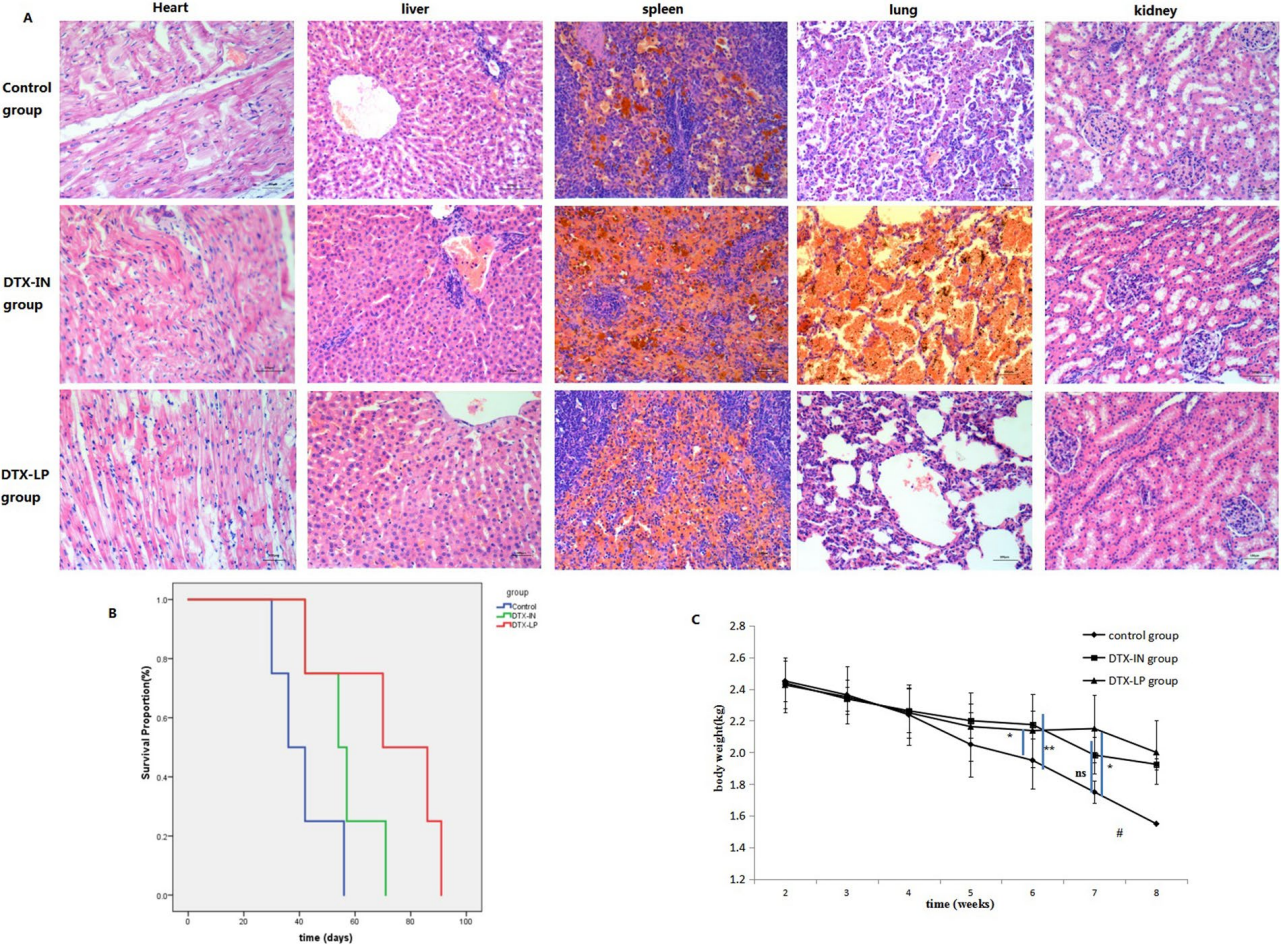
Survival analysis in VX2 orthotopic lung cancer rabbits with treatment of saline, DTX-IN and DTX-LP after six times administrations (**A**) Histological evaluation of major organs (×40) (heart, liver, spleen, lung and kidney); (**B**) Kaplan-Meier survival curves (n = 4) *p < 0.05, **p < 0.01, “ns” means no significant difference; (**C**) The body weight change of VX2 orthotopic lung cancer rabbits during treatment. Results are means ± SD, n = 4. #no SD because of death in control group, *p < 0.05, **p < 0.01, “ns” means no significant difference.

Kaplan-Meier survival curves indicate a statistically significant survival advantage of the DTX-LP group over DTX-IN or control groups (Fig. 9B). The average survival days of the control, DTX-IN, and DTX-LP groups were 41.0 ± 11.1, 56.0 ± 11.9, and 92.3 ± 21.8 days, respectively. HE staining of major organs (lungs, liver, kidneys, spleen and heart) was used to evaluate the tumor metastasis and organ toxicity associated with the different DTX formulations (Fig. 9A). In contrast to the control group, there was no noticeable tumor metastasis in the DTX-IN or DTX-LP groups. In the control group, the tumor spread to the right lung and showed pleural metastases. Many acidophilic leukocytes and macrophages were visible with distorted lung architecture. Dilation of the splenic sinus, glomerulus pyknosis, and local hepatocyte swelling were observed in the DTX-IN group only. These histological changes indicate that DTX-IN exhibited greater toxicity than DTX-LP due to the systemic distribution of DTX after intravenous administration of DTX-IN compared to DTX-LP. There were no noticeable histological changes in the DTX-LP group, indicating no organ toxicity. Changes in body weight were observed throughout the survival time across all groups (Fig. 9C). Food intake of rabbits in the control group was reduced and the body weights, likely due to rapid tumor growth, and metastasis. The average weight loss rate of the control group after six weekly administrations was 32.6%. The DTX-IN and DTX-LP groups did not show the extent of weight loss as the control group. The average weight loss rates of the DTX-IN and DTX-LP groups after six weekly administrations were 25.2% and 15.6%, respectively.

### DTX and its metabolites in feces

**Figure 10:**
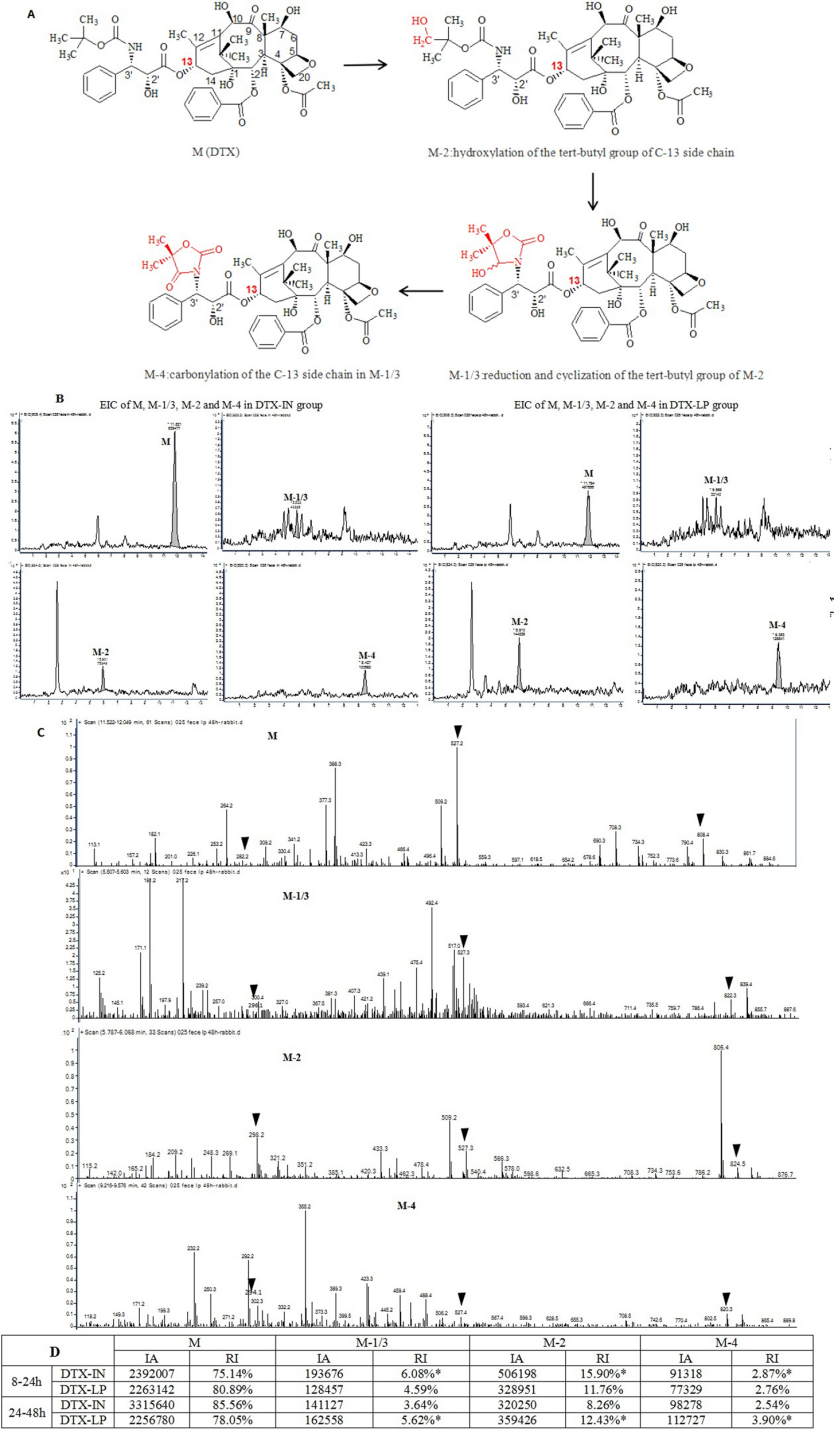
DTX and its metabolites in feces measured by UPLC-MS/MS. (**A**) DTX (M) and its known metabolites M-1/3, M-2, M-4 *in vivo*. (**B**) The extracted ion chromatogram (EIC) of M-1/3, M-2, M-4 were found both in DTX-LP and DTX-IN groups (the MH^+^ at m/z 808, 822, 824 and 820); (**C**) The second order mass spectrum of DTX and M-1/3, M-2, M-4 in DTX-LP group (▾ means the protonated parent ion and ion fragments); (**D**) The iron abundances and relative iron intensity during 8–24 h and 24–48 h in DTX-LP and DTX-IN groups (IA: the iron abundances; RI: the relative iron intensity, RI(x) = IA(x)/IA(M) + IA(M-1/3) + IA(M-2) + IA(M-4), x means M, M-1/3, M-2 or M-4; *means the peak value).

DTX and its known metabolites M-1/3, M-2, and M-4 (Fig. 10A) were found in the feces of both in DTX-LP and DTX-IN groups with the protonated parent ion (MH^+^) at m/z 808, 822, 824 and 820, respectively. The retention times of DTX, M-1/3, M-2 and M-4 were 11.8 min, 5.5 min, 5.9 min, and 9.4 min (Fig. 10B, extracted ion chromatogram, EIC). All four substances revealed a characteristic fragment at m/z 527 called a baccatin structure. For DTX, the MH^+^ typical ion typical fragments at m/z 527 and m/z 282, representing cleavage of the C-13 side chain, were shown in the second-order mass spectrum. For M-2, the MH^+^ ion fragments at m/z 527 and at m/z 298, reflecting the hydroxylation of the tert-butyl group of the C-13 side chain, were shown in the second-order mass spectrum. The enantiomers M-1 and M-3, showed characteristic ions at m/z 527 and m/z 296 in the second-order mass spectrum, which represent the C-13 side chain after reduction and cyclization of the tert-butyl group of M-2. For M-4, the MH^+^ ion fragments at m/z 527 and at m/z 294 reflect the carbonylation of the C-13 side chain in M-1/3 (Fig. 10C, the second order mass spectrum of DTX and M-1/3, M-2, M-4). Spectra from the DTX-LP group samples showed no significant differences in the metabolism pathway or metabolites when compared to samples from the DTX-IN group. According to the iron abundances of M, M-1/3, M-2 and M-4, we compared the temporal metabolism of DTX in DTX-LP and DTX-IN groups over three different time periods (0–8 h, 8–24 h and 24–48 hours). The results confirm that metabolites of DTX reached peak concentration levels during the 8–24 h sampling period in the DTX-IN group and during the 24–48 h sampling period in the DTX-LP group (Fig. 10D). Additionally, several unknown metabolites with the protonated parent ion (MH^+^) at m/z 839 were found in both the DTX-LP and DTX-IN groups. It is worth defining the structure of these unknown metabolites in future work.

## Discussion

DTX is a conventional chemotherapeutic of choice for many cancers including NSCLC, but its effectiveness in lung cancer therapy is limited due to significant side effects resulting from toxicity after intensive therapy. To enhance the antitumor efficacy in lung cancer and reduce the systemic side effects, we designed a novel formulation, DTX-LP. DTX-LP offers both increased antitumor efficacy and reduced toxicity compared to DTX-IN since more DTX can be delivered directly into intrapulmonary tumors after intravenous injection of DTX-LP compared to DTX-IN. In our previous study, the preparation of DTX-LP was expounded in detail. The purpose of this study was to investigate the targeting efficiency and antitumor activity of DTX-LP against DTX-IN *in vivo*.

The evaluation of antitumor activity *in vivo* was based on tumor-bearing animals. To date, the tumor-bearing nude or immunodeficient mouse model of lung cancer has been extensively applied for pharmacodynamic studies to establish and observe tumor growth for its convenience. In this model, the tumor is usually propagated through a subcutaneous xenograft^25, 26^, which does not grow not in lungs. It has been reported that endobronchial/intrapulmonary injection has been used to establish a mouse model of lung cancer^27, 28^. However, this method has been not used widely due to the small size of mouse lungs and other technical difficulties. The subcutaneous xenotransplanted tumor model in nude mice cannot accurately assess the antitumor activity of DTX-LP, an organ-targeted formulation that accumulates in the lungs. Therefore, it was necessary to establish an orthotopic lung cancer model to study the antitumor activity of DTX-LP.

The VX2 lung cancer rabbit model has been an ideal model that shows orthotopic, aggressive, and metastatic growth. VX2 tumor cell lines stemmed from the squamous cell carcinoma derived from the papilloma induced by Shope-papilloma virus. Previous methods reported for establishing a VX2 lung cancer rabbit model include percutaneous injection of a VX2 tumor cell suspension with/without CT^29, 30^ and implantation of VX2 tumor tissue fragment under traditional thoracotomy^31, 32^. However, the former method may result in pleural dissemination and multifocal growth. The latter easily leads to pneumothorax, which requires the surgeon to have considerable proficiency and experience. The minimally invasive percutaneous puncture inoculation to establish a VX2 orthotopic lung cancer rabbit model in our study overcame the limitations of the above two methods with fewer technical difficulties, decreased mortality of rabbits, and higher success rate. The antitumor effect of DTX-LP was evaluated based on this orthotopic lung cancer model for the first time.

At present, the key issue for lung cancer therapy is how to prepare an appropriate treatment that will increase the concentration of antitumor drugs targeted to tumors. Delivery of a higher load of antitumor drug inside the tumor translates into significantly increased antitumor activity *in vivo* ^33^. Our study confirms that DTX-LP significantly accumulates in both the lung tissue and tumor by HPLC analysis. The accumulation of DTX in intrapulmonary tumors was attributed to active distribution of liposomes in lung and passive EPR effect of tumor tissues. The active distribution of DTX in lungs might be related to negative potential of DTX-LP. According to the preliminary experiments, it is probable that the negative charge of DTX-LP reversibly and noncovalently is adsorbed on the surface of red blood cells (RBC) in the blood stream, and then detached likely due to shear or direct RBC-endothelium contact during their passage through the lung tissue microvasculature. The detached DTX-LP interacts with lung cells, such as adsorption, lipid exchange, endocytosis and fusion. The EPR effect, in which the leaky vasculature of the tumor environment provides greater permeability, is the major mechanism for delivering DTX to tumor tissues. The current results suggest that the concentration of DTX in tumor tissue after treatment with lung-targeted DTX-LP is significantly higher than that after treatment with the non-lung-targeted DTX-IN formulation.

The increase of DTX concentration in tumors enhances the antitumor effect after treatment with DTX-LP based on the VX2 orthotopic lung cancer rabbit model in our study. *In vivo* CT imaging is an important tool for monitoring the change of tumor size^34^. It offers a continuous series of images without sacrifice of tumor-bearing rabbits before and after treatment by different formulations. Our conclusions regarding tumor volumes and TIRs are based on CT imaging. Compared to the DTX-IN and control groups, animals treated with DTX-LP had smaller tumor volumes. TIRs in the DTX-LP group were significantly higher than those in the DTX-IN group. Flow cytometry and TUNEL assay are common methods used to monitor tumor apoptosis^35, 36^. DTX-LP increased the apoptotic index compared to DTX-IN, suggesting that DTX-LP increases apoptosis of tumor cells, thereby reducing tumor growth. The results from immunohistochemistry of PCNA and CD31 in tumor tissues are consistent with the tumor apoptosis data. The PCNA-positive ratio reveals tumor proliferation^36^. CD31, a widely used immunohistochemical marker of endothelial cells, is expressed in both proliferating and resting endothelial cells^37^. CD31-positive expression by immunohistochemistry is an indicator of neovascularization in tumor tissues. Inhibition of tumor angiogenesis can lead to tumor inhibition. The DTX-LP group had weaker PCNA and CD31 signals compared to the DTX-IN groups, suggesting that lung-targeted liposomes successfully delivered DTX to the lung tumor tissue to inhibit tumor growth, reduce tumor mass and enhance antitumor effects.

The accumulation of DTX in lung and tumor tissues after treatment with DTX-LP partly reduced the concentration of DTX in other tissues, such as liver, spleen, and kidney. The concentrations of DTX in liver (in μg DTX/g tissue) at 30 min, 1.5 h, 4 h, 8 h, and 12 h after treatment with DTX-LP were 4.77 ± 0.33, 5.87 ± 3.01, 5.80 ± 1.03, 4.98 ± 0.4, and 1.71 ± 0.49 μg/g, respectively. Liver concentrations at the same time points in the DTX-IN group were 6.05 ± 0.78, 6.22 ± 0.60, 7.41 ± 0.20, 5.70 ± 0.93, and 3.53 ± 0.38 μg/g, respectively. The concentrations of DTX in kidney at 30 min, 1.5 h, 4 h, 8 h, and 12 h after treatment of DTX-LP were 4.58 ± 1.00, 7.93 ± 1.03, 6.44 ± 1.76, 2.57 ± 0.10, and 4.27 ± 0.55 μg/g, respectively. Kidney concentrations at the same time points in the DTX-IN group were 7.88 ± 0.73, 8.16 ± 0.75, 9.64 ± 0.87, 3.88 ± 0.79, and 8.69 ± 0.41 μg/g, respectively. The concentrations of DTX in spleen at 30 min, 1.5 h, 4 h, 8 h, and 12 h after treatment of DTX-LP were 5.19 ± 2.20, 4.91 ± 0.17, 7.22 ± 1.10, 8.99 ± 1.97, and 5.97 ± 1.13 μg/g, respectively. Spleen concentrations at the same time points in the DTX-IN group were 6.51 ± 0.66, 8.29 ± 1.65, 13.74 ± 0.72, 9.88 ± 1.34, and 7.61 ± 0.48 μg/g, respectively. The similar phenomenons that the concentration of DTX in DTX-LP group was lower than DTX-IN group were found in the major organ tissue, including liver, spleen and kidney. The reduced concentrations of DTX in these organs in the DTX-LP group compared to the DTX-IN group may lead to reduced organ toxicity, also suggested by HE staining^38^. In DTX-IN group, HE staining indicated that swollen liver cells, splenic sinus dilation with more hematoxin granules and glomerular consolidation which might relate to higher concentrations of DTX. However, there was mild pathological changes in DTX-LP group. The results of HE staining were consistent with the limited organ distribution of DTX in the DTX-LP group compared to the DTX-IN group.

The main purpose of DTX injection for NSCLC is to prevent tumor metastasis and recurrence, prolong survival, and improve quality of life^39^. The survival state is an important index to evaluate antitumor efficacy in animal models of lung cancer. The survival state includes survival time, tumor metastasis, change of body weight, food availability and so on. According to the results, rabbits in control group were died of respiratory failure with significant weight loss. The anatomy confirmed that the tumor had been transferred to the chest and mediastinal. The results indicate that DTX-LP prevents tumor metastasis to the chest wall, prolongs the average survival time by twice as long, and improves the condition of weight loss.

DTX is mainly metabolized in the liver and excreted in the feces. The main metabolite formed in rabbits is hydroxylated at C13 of the tert-butyl side chain and further oxidized and cyclized. DTX-LP alters the bioactivity of DTX with an effect of increased lung and tumor targeting. The added liposome may lead to changes in metabolic behavior. Although there was no significant difference in metabolism between DTX-LP and DTX-IN, liposomal delivery of DTX significantly delayed the drug metabolism in rabbit feces compared to the traditional injection formulation. This likely results in a prolonged accumulation of DTX in the lungs and tumors, helping the drug to fully exert its cytotoxic activity against NSCLC. Delayed metabolism of DTX-LP may be evidence for enhanced therapeutic efficacy and reduced systemic toxicity.

In summary, the DTX lung-targeted liposome formulation enhances the antitumor effect of DTX and reduces the systemic toxicity of the drug. DTX-LP is a promising formulation for targeted therapy of lung cancer.

## Methods

### Ethics statement

This study was approved by the Ethics Committee of Chongqing Medical University to ensure humane treatment of test animals and all experiments were performed in accordance with relevant guidelines and regulations.

### The characterization of DTX-LP

The zeta potential and size distribution of DTX-LP was determined by Malvern laser particle size analyzer (Nano ZEN3600). The encapsulation efficiency of DTX-LP was determined by HPLC assay (Agilent 1100 with UV detector). The mobile phase was a mixture of acetonitrile and water (50:50, v/v) delivered at a flow rate of 1 ml/min. The wavelength used for the study was 230 nm.

### Minimally invasive percutaneous puncture inoculation to establish the VX2 orthotopic lung cancer rabbit model

New Zealand White rabbits (Chongqing Medical University), male or female, weighing between 2.0 and 3.0 kg, were used and allowed free access to food and water before inoculation.

Rabbits for inoculation were anesthetized by intravenous injection of pentobarbital sodium (3%, 1 ml/kg, Ourchem®, Shanghai) and fixed on the digital gastrointestinal machine (Fig. 1A, UD150 L-30E, Shimadzu). The right chest regions were shaved and disinfected. Percutaneous puncture inoculation of VX2 tumor fragments was divided into multiple steps: First, an 18-gauge needle containing VX2 tumor fragments and gelatin sponge was placed over the region of the right chest of a rabbit. The point, angle, and depth of puncture were determined with imaging features of the digital gastrointestinal machine (Fig. 1B). Next, each rabbit received a percutaneous puncture though the right chest by the 18-gauge needle. The position of the needle *in vivo* was determined using the digital gastrointestinal machine (Fig. 1C). When the needle point was inserted into right lung of a rabbit, the VX2 tumor fragment and gelatin sponge were pushed into the right lung together. Finally, after withdrawing the needle, pressure was applied to the puncture point for 90 seconds (Fig. 1D). After the inoculation, an intramuscular injection of cefradine (0.25 g/d, North China Pharmaceutical Group Co., Ltd) was given to each rabbit to prevent infection.

### Treatment administration schedule

Forty-eight VX2 orthotopic lung cancer rabbits were randomly divided into three groups: a control group (n = 6), DTX-IN group (n = 21), and DTX-LP group (n = 21). Three groups of tumor-bearing rabbits received an intravenous injection of saline once a week, DTX (Texotere®, 1 mg/kg, once a week) and DTX-LP (prepared in our own laboratory^22^, 1 mg/kg, once a week) beginning at two weeks post-inoculation.

### CT imaging and TIR

Rabbits in each group were randomly selected to receive CT scans (Aquilion CXL, TOSHIBA) before administration and after the third and sixth administrations. The rabbits were anesthetized by intravenous injection of pentobarbital sodium (3%, 0.5 ml/kg) before CT scanning. The scanning parameters were 80 Kv, 30 mA, 5 mm slice thickness; pulmonary window: 1200 HU window width, −400 HU window level; mediastinal window: 300 HU window width, 25 HU window level. Tumor length, width and thickness were determined to calculate the tumor volume according to the following formula:

$${\rm{V}}({{\rm{mm}}}^{3})={\rm{L}}({\rm{mm}})\times {{\rm{S}}}^{2}({{\rm{mm}}}^{2})/2$$

where V is tumor volume, L is the largest diameter (the maximum value of tumor length, width, and thickness) and S is the smallest diameter (the minimum value of tumor length, width, and thickness).

TIRs were calculated according to the following formula:

$${\rm{TIR}}=\frac{({{\rm{V}}}_{2}-{{\rm{V}}}_{1})}{{{\rm{V}}}_{2}}\times 100 \% $$

where V_2_ is tumor volume in control group (mm^3^) and V_1_ is tumor volume in DTX-IN or DTX-LP groups (mm^3^).

### HPLC assay to detect DTX distribution in tumors

Tumor-bearing rabbits in the DTX-IN and DTX-LP groups were sacrificed at different time points, including 30 min, 1.5 h, 4 h, 8 h and 12 h after the last treatment administration (three rabbits at each time point, n = 3). The tumor and major organs including heart, liver, spleen, lungs, kidneys, stomach, and brain were dissected out for DTX level analysis by HPLC. All tissues were cut into pieces after being washed with cold PBS solution (pH 7.2–7.4) and homogenized with twice the amount of purified water (g:ml). Then, 100 μl of internal standard (paclitaxel, 10 μg/mL) and 3 ml mixed solution (methanol: 0.03% ammonia, 4:1, v/v) were added to each homogenate sample. The samples were then vortexed for 5 min and centrifuged at 15,000 rpm for 10 min. The upper layers were removed into the activated solid phase extraction columns. Then, the columns were eluted by 6 ml purified water followed by 3 ml mixed solution (methanol: 0.03% ammonia, 3:2, v/v), 3 ml mixed solution (methanol: 0.03% ammonia, 2:3, v/v), and 2 ml methanol. The last elutions with 2 ml methanol were collected into clean tubes and dried under nitrogen stream at 40 °C. The residue was dissolved in 200 μl methanol for further HPLC analysis. All samples were analyzed for DTX concentration by reversed-phase HPLC in an Agilent 1100 HPLC system with UV detector. The column was a Phenomenex Luna (250 mm × 4.6 mm) column, 5 μm particle size, and the mobile phase was a mixture of acetonitrile and water (55:45, v/v) delivered at a flow rate of 1 ml/min. The wavelength used for the study was 230 nm.

### Necropsy and Tissue Preparation

Tumor-bearing rabbits (except those used for the HPLC assays) in each group were sacrificed after the last administration or natural death, and the daily behavior, such as weight and breathing, were recorded during the survival time. All of these animals were dissected after sacrifice or natural death. The lung, tumor, heart, liver, kidney and spleen were grossly examined and saved. For HE staining procedures and immunohistochemical analyses (TUNEL, PCNA and CD31), one part of the tumor was fixed in formalin and embedded in paraffin. Another part of the tumor was washed in cold PBS and later used for flow cytometry. The other tissues were fixed in formalin and embedded in paraffin for pathology tests.

### Immunohistochemistry by TUNEL assay and tumor proliferation assay

Tumor apoptosis was measured using a TUNEL assay. The paraffin sections (5μm) were dewaxed with xylene and gradient concentrations of ethanol. Then, they were washed with PBS (pH 7.2–7.4, Beijing Jinshan Biotechnology Co., Ltd) three times. The sections were incubated in 3% H_2_O_2_ and proteinase K (Merck Millipore), reacted with TUNEL (in situ cell death detection kit, POD method, Roche Group) mixture for 1 h. After washing with PBS, the sections were incubated with streptavidin-HRP (1:400) for 30 min at 37 °C and rewashed with PBS. Then, the sections were incubated with DAB solution (Beijing Jinshan Biotechnology Co., Ltd) for 10 min. Finally, counterstaining was performed with hematoxylin (Beijing Jinshan Biotechnology Co., Ltd). Afterwards, the tumor slides were imaged under a microscope. Apoptosis was quantified as the percent of TUNEL-positive cells relative to the total cell number. Proliferating tumor cells were identified using an antibody against the PCNA (monoclonal antibody, Immunoway®). The procedure was performed as above described with minor modifications. Briefly, PCNA antibody was incubated as the primary antibody. The number of PCNA-positive cells was measured under a microscope. Cell proliferation was calculated as the percent of PCNA-positive cells relative to the total cell number.

### Flow cytometry

The tumors, which had been washed in cold PBS, were cut into 0.1 mm^3^ pieces. Next, the tumor pieces were sifted through a 200-mesh cell strainer to remove the cell mass and debris. The cell suspensions after sifting were centrifuged at 1,000 rpm and resuspended in cold PBS. The tumor cells were stained with annexin V-FITC and PI. Flow cytometry was performed to analyze apoptosis according to manufacturer’s instructions. Approximately 10^4^ events (cells) were evaluated for each sample.

### Tumor angiogenesis by CD31 assay

Tumor angiogenesis was measured with immunohistochemistry by CD31 assay. Briefly, the paraffin sections were dewaxed with xylene and washed with PBS (pH 7.4) three times. Prior to the application of the primary antibody, normal goat serum was incubated with the sections to block the non-specific protein binding sites for 20 min. Then, a mouse anti-CD31 monoclonal antibody (Immunoway®) was incubated with the sections as the primary antibody for detecting CD31. After 1 h incubation at 37 °C, the slides were rinsed with PBS three times and incubated with goat anti-mouse antibody as the secondary antibody. After 15 min incubation at 37 °C, the slides were rinsed with PBS three times and incubated with DAB solution. Finally, counterstaining was performed with hematoxylin for microscopic observation.

### Docetaxel and its metabolites in feces

Feces from the three groups were collected during the time periods 0–8 h, 8–24 h, and 24–48 h after first drug administration to trace the DTX and its metabolites with ultra-performance liquid chromatography (UPLC, Agilent 1290) and tandem mass spectrometry (MS/MS, Agilent 6460). The feces samples were homogenized 1:4 (g/v) in 4% of bovine serum albumin in water (g/v). The feces homogenates were extracted twice with diethyl ether (1:5, g/v). After vortexing for 30 min and centrifugation for 5 min at 15,000 rpm, the diethyl ether layers were removed and evaporated at 37 °C under nitrogen. The residues were redissolved in 200 μl methanol and subjected to UPLC-MS/MS analysis. Gradient separation was performed using mobile phase A (formic acid/water, 1:1000, v/v) and mobile phase B (methanol/acetonitrile, 20/80, v/v). Initial conditions were 55% A and 45% B from 0 to 14 min. From 14 to 20 min, a linear gradient was applied to a final ratio of 0% A and 100% B, which was maintained for another 5 min. Positive mass spectra were recorded for each sample.

### Statistical Analysis

Descriptive statistics include the mean and standard error and were determined with Microsoft Excel 2010. The statistical analysis was performed with SPSS 23.0 software (SPSS, IBM Inc.). The statistical significances of differences between treatment groups in tumor growth inhibitory efficacy, apoptosis, and cell proliferation were calculated using the Student’s t test. Kaplan-Meier survival curves used the LOG-RANK test. All *p* values less than 0.05 were considered significant.

## Electronic supplementary material

The supplementary file

Dataset1
