# Supplementary material for: Enhanced antitumor effect on intrapulmonary tumors of docetaxel lung-targeted liposomes in a rabbit model of VX2 orthotopic lung cancer
Source: Sci Rep. 2017 Aug 30;7:10069. doi: 10.1038/s41598-017-10530-8 (PMC5577178; doi:10.1038/s41598-017-10530-8)
Supplement: Supplementary file 1 — The supplementary file [file 41598_2017_10530_MOESM1_ESM.pdf]

Enhanced antitumor effect on intrapulmonary tumors of docetaxel lung-targeted liposomes in a rabbit model of VX2 orthotopic lung cancer

LiJuan Wang[1,2], Rui Li[1], KeKe Che[3], ZhongHong Liu[1], ShiFeng Xiang[4], MengYa Li[1] & Yu Yu[1]*

1. Pharmacy College, Chongqing Medical University, Chongqing 400016, China; 2. Department of Pharmacy, Chongqing Medical and Pharmaceutical college, Chongqing 401331, China; 3. Department of Pharmacy, Chongqing General Hospital, Chongqing 400014, China; 4. Radiology department, Chongqing General Hospital, Chongqing 400014, China

*Corresponding author. E-mail: yuyu3519@163.com

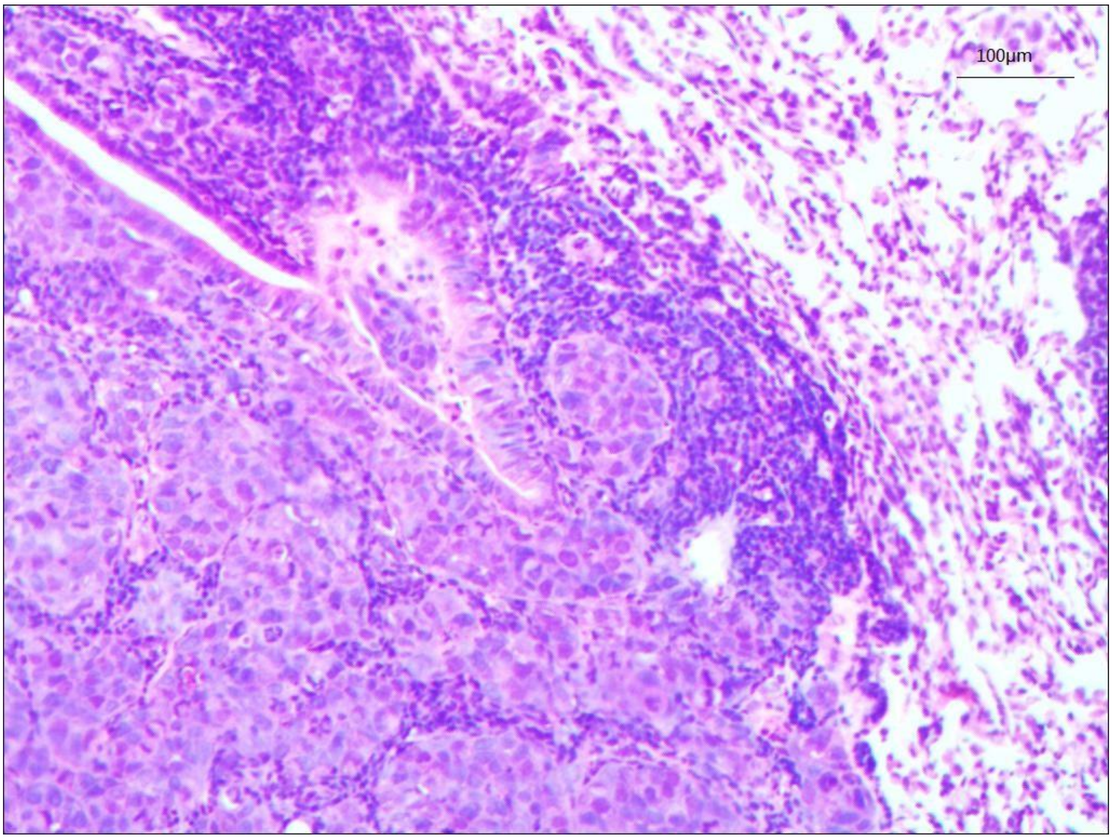

Fig.3C-1 in revised manuscript (the original images)

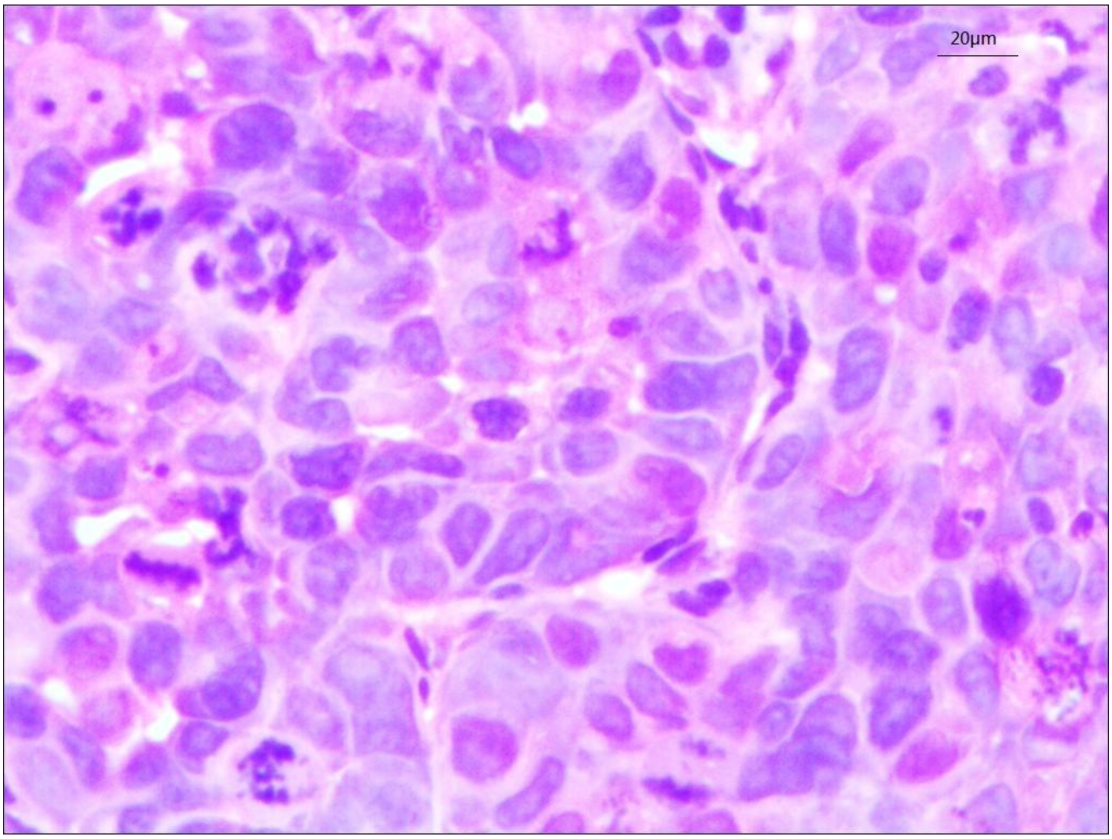

Fig.3C-2 in revised manuscript (the original images)

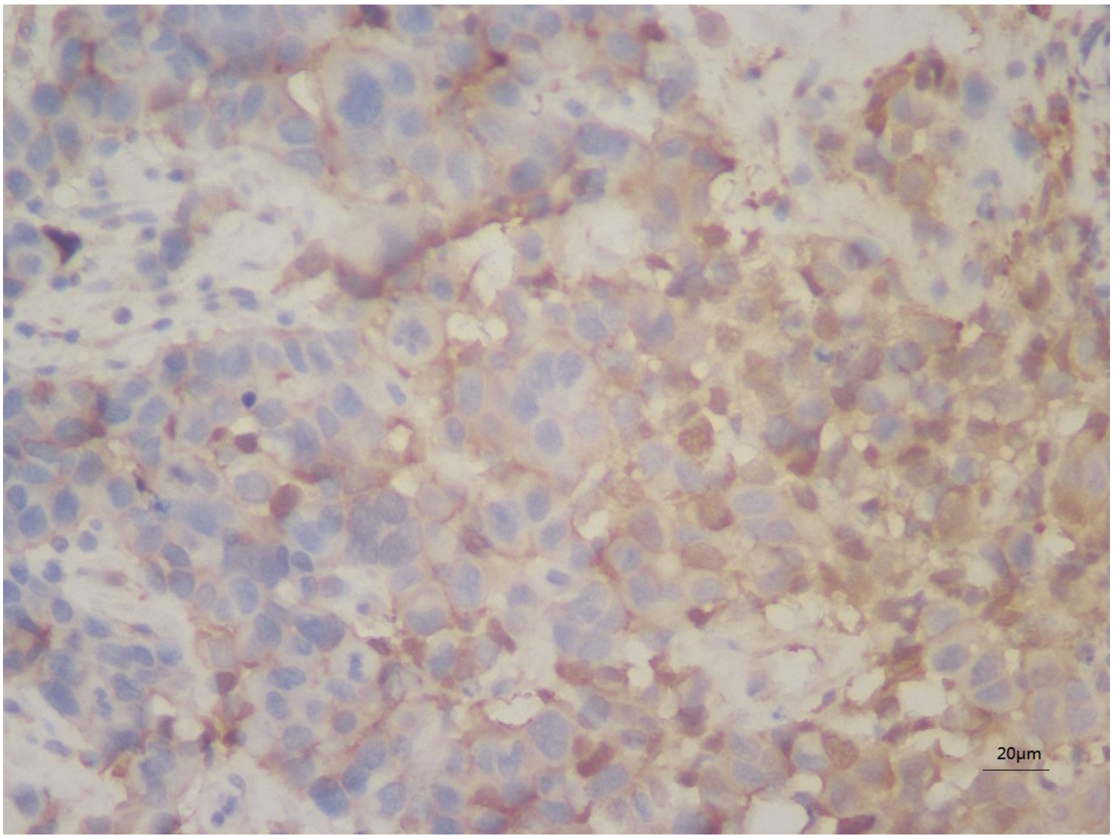

Fig.7A in revised manuscript (the original images)

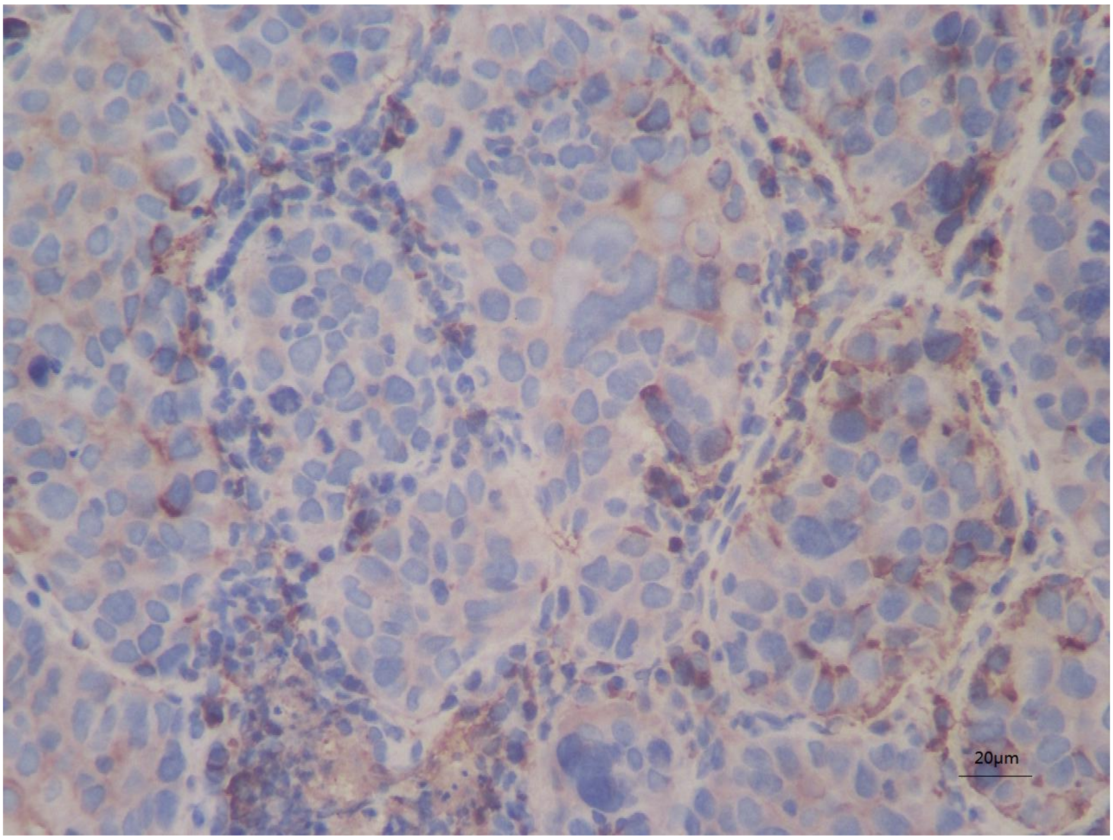

Fig.7B in revised manuscript (the original images)

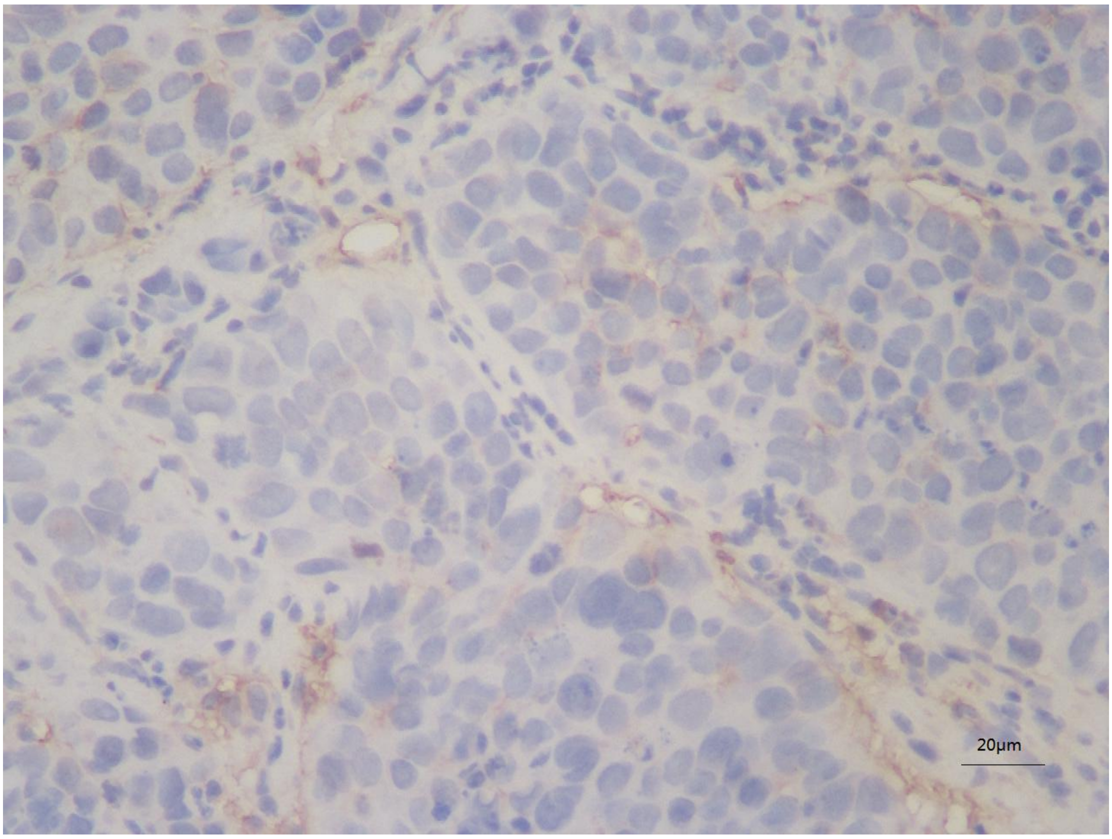

Fig.7C in revised manuscript (the original images)
